# Editorial: Nutrient dependent signaling pathways controlling the symbiotic nitrogen fixation process, Volume II

**DOI:** 10.3389/fpls.2023.1210114

**Published:** 2023-05-29

**Authors:** Vladimir Totev Valkov, Maurizio Chiurazzi

**Affiliations:** Institute of Biosciences and Bioresources (IBBR), Italian National Research Council (CNR), Napoli, Italy

**Keywords:** ureides, ascorbic acid, small heat shock proteins, ROS, biotic and abiotic stress

Symbiotic Nitrogen Fixation (SNF) is a vital process in agriculture since it allows for atmospheric nitrogen assimilation through legume-rhizobia symbiosis. This process sustains plant growth on nitrogen-poor soils and legumes are utilized in crop rotation cultivations for limiting N-fertilizers use, resulting in enhanced plant growth. SNF is also based on a complex relationship between the host-bacteria symbiosis and environment. The symbiosis can be affected by a number of abiotic constrains as soil composition (including nutrient availability), salinity and especially drought. Moreover, in the recent decades, considerable progresses have been made to explore novel genes controlling the symbiotic signaling pathways and nodule functioning in legume plants (Mergaert et al., 2020). The aim of the volume II of this Research Topic was to present new findings in these scientific field of study.

The five research articles included in the volume presents new original data for four agronomically important legume plants: soybean (*Glycine max* L.), faba bean (*Vicia faba* L), white clover (*Trifolium repens*) and *Medicago truncatula*. In all of them different aspects of the process of SNF were discussed and different biotic and abiotic factors influencing this process were investigated. In addition, all articles pointed on the practical application of the obtained knowledge for sustainable agriculture, environmental safety and economic competitiveness.

Ureides (allantoin and allantoic acid) are the primary transport forms for fixed-N in soybean, and the concentration in plant tissue correlates with the fraction of N derived from N_2_-fixation (Herridge and Peoples, 2002). Therefore, the relative abundance of ureides (RAU) can be used to quantitatively assess N-fixation (Unkovich and Pate, 2000). In order to enable the RAU predictions as a measure of the fraction of N derived from N-fixation, de Borja Reis et al. analyzed in a complex study, how different environmental covariables such as N fertilization, atmospheric vapor pressure deficit, precipitation, sowing date, drought stress and other factors may affect the correlation between RAU and N_2_-fixation. Based on large amount of data collected from 21 field experiments conducted at 11 research stations in six US states, authors created a model in which some expected association between RAU and environmental conditions were observed and new relationships for future investigations were proposed.

As discussed in de Borja Reis et al. the N-fixation process is highly susceptible to drought conditions. The regulation of SNF under drought involves diverse factors as internal oxygen availability, carbon limitation, and N-feedback regulation. Proteomic analysis suggests that plant carbon metabolism, protein synthesis, and cell growth are among the processes most altered in soybean nodules under drought stress (Gil-Quintana et al., 2013). The obtainment of root and nodule transcriptome, proteome and metabolome maps in relation to drought, including in-depth functional characterization of transcripts/proteins/metabolites and how they lead to better drought tolerance are extremely important for the development of tools aimed to overcome the drought stress (Kunert et al., 2016). One of the effects of drought stress in plants is an overproduction of reactive oxygen species (ROS), leading to oxidative damage at the cellular level and, ultimately, cell death (Cruz De Carvalho, 2008). Plants are able to counteract such damage via the production of antioxidant compounds such as ascorbic acid (AsA) (Foyer and Noctor, 2011). In this Research Topic, Cobos-Porras et al. hypothesized that increased AsA pools could lead to improved SNF under stressful drought conditions. To explore this model authors generated stable transformed *Medicago truncatula* plants overexpressing *MtVTC2*, a gene encoding for GDP-L-galactose phosphorylase. Overexpression of *MtVTC2* increased the total AsA in transgenic plants. Surprisingly, the increased AsA availability did not provide an advantage in terms of plant growth or symbiotic performance either under well-watered conditions or in response to drought. As discussed by the authors the failure of this mere constitutive overexpression strategy could be due to the involvement of antioxidant compounds such as AsA in a variety of metabolic pathways where they play complex regulatory roles. Therefore, a possible tuning for the improvement of the strategy could be related to the exploitation of nodule specific promoters to drive the overexpression only in the N_2_-fixing zone of the nodule.

Additionally, small heat shock proteins (sHSPs) are largely involved in the response to different environmental stresses including drought. sHSPs are present in all organisms and involved in multiple plant developmental processes, but their role in nodule development in soybean is largely unknown (Yang et al., 2021). In this Research Topic, Yang et al. investigated the role of one sHSP, GmHSP17.1 in the process of soybean nodulation and N_2_-fixation. An in-depth molecular and biochemical characterization of transgenic soybean plants with either overexpressed or suppressed (by RNA interference) *GmHSP17.1* was carried out. *GmHSP17.1* overexpression promoted an increase of nodules number and size, and nitrogenase activity, whereas RNAi lines showed significantly impaired nodule development and N_2_-fixation. Furthermore, a direct interaction of GmHSP17.1 with the peroxidase GmRIP1 was demonstrated through liquid chromatography-tandem mass spectrometry, yeast-two hybrid, and bimolecular fluorescence complementation. *GmRIP1* is preferentially expressed in soybean nodules where appears to be a direct target of *GmHSP17.1*. In fact, the ROS content greatly decreased in *GmHSP17.1* overexpression lines and increased in suppression lines. These results could be very useful for genetic improvement of legume plants in the future.

Another study presented in this Research Topic with the prospect of direct applications in sustainable agriculture is the one reported by Weith et al. In order to improve the quality of New Zealand pastures by limiting the application of N fertilizers, they have investigated quantitative parameters associated to genetic variations of white clover (*Trifolium repens L.)* families under symbiotic and non symbiotic conditions. The establishment of a vigorous growth of white clover in the first year is a critical quantitative parameter for high quality mixed pastures. A genetically structured breeding population comprising 120 half-sibling families was assessed for symbiotic traits with a commercial *Rhizobium* strain (TA1). Quantitative genetic analysis identified significant family additive genetic variance for dry shoot and roots, symbiotic potential, and root to shoot ratio. Although moderate correlations have been found between the phenotypic parameters analyzed and families genetic variations, the obtained results provide a promising platform to breed white clover with improved symbiotic traits and are informative for other legume crops.

In the recent years application of beneficial rhizosphere microbiota as an alternative strategy to chemical treatments to enhance plant performances and to increase resistance to biotic and abiotic stresses has become a promising option for agriculture (Rizvi et al., 2022). In this Research Topic, Abdelkhalek et al. focused their research on nitrogen-fixing plant growth-promoting rhizobacteria (PGPR), *Rhizobium leguminosarum* bv. *viciae* strain 33504-Alex1, isolated from the root nodules of faba bean. The applications of 33504-Alex1 in faba bean plants through either soil or foliar treatments, promoted a considerable reduction in disease incidence and severity of *Bean Yellow Mosai Virus* (BYMV) infection, resulting in an improved growth and increased total chlorophyll content. The treatment with 33504-Alex1 determined a reduction of non-enzymatic oxidative stress markers associated to the increase of free radicals and reactive oxygen species scavenging activities as well transcriptional levels of pathogenesis-related genes. Furthermore, high-performance liquid chromatography and gas chromatography-mass spectrometry analyses indicated an accumulation of polyphenolic compounds as likely elicitor of an induced systemic acquired resistance in the treated faba bean plants. As a result, PGPR-Alex1 can be used as a simple, environmentally safe, bio-fertilize/bio-controller to protect faba bean plants from BYMV infection.

In summary the five articles included in this Research Topic provide new inside knowledge in specific aspects of the SNF. The important role of GmHSP17.9 in this process and the missing effect of AsA on the symbiotic performance are intriguing results. The positive effect of PGPR 33504-Alex1 for an efficient biocontrol and the models developed after analyses of the effect of environmental factors or quantitative genetic on SNF will be of future interest for sustainable agriculture.

## Author contributions

All authors listed have made a substantial, direct, and intellectual contribution to the work and approved it for publication.
